# Prevalence and Pattern of Congenital Anomalies Among Deliveries and Pregnancy Terminations at a Tertiary Care Center

**DOI:** 10.7759/cureus.112175

**Published:** 2026-07-06

**Authors:** Ravindra S Pukale, Greeshma V R

**Affiliations:** 1 Obstetrics and Gynaecology, Adichunchanagiri Institute of Medical Sciences (AIMS), Adichunchanagiri University, B.G. Nagara, IND

**Keywords:** congenital anomalies, pregnancy outcomes, prenatal ultrasonography, prevalence, tertiary care centre

## Abstract

Introduction: Congenital anomalies are an important cause of perinatal morbidity and mortality, particularly in low- and middle-income countries. Data on their prevalence and pattern remain limited because many studies exclude stillbirths and medically terminated pregnancies. This study aimed to determine the prevalence and pattern of congenital anomalies and describe the maternal demographic and obstetric characteristics, mode of anomaly detection, and pregnancy outcomes at a tertiary care center.

Materials and methods: This prospective observational study was conducted in the Department of Obstetrics and Gynaecology, Adichunchanagiri Institute of Medical Sciences, B.G. Nagara, Karnataka, India, from February 2025 to January 2026. All deliveries, including live births, stillbirths, and medically terminated pregnancies with documented fetal congenital anomalies, were screened. Maternal demographic and obstetric characteristics, antenatal findings, congenital anomaly patterns, mode of detection, and pregnancy outcomes were recorded. Data were analyzed using descriptive statistics and expressed as frequencies and percentages.

Results: Among 1,878 deliveries and medically terminated pregnancies screened, congenital anomalies were identified in 47 (2.5%) cases. Central nervous system anomalies were the most common, followed by circulatory and gastrointestinal anomalies. Most anomalies were detected antenatally by ultrasonography (37 (78.7%)). Medical termination of pregnancy was the most frequent outcome (31 (66.0%)), followed by stillbirth (10 (21.3%)) and live birth (6 (12.8%)). Most affected pregnancies occurred among women aged 26-30 years, multigravida mothers, and those diagnosed between 21 and 25 weeks of gestation. Previous spontaneous abortion was the most frequently observed maternal characteristic.

Conclusion: Congenital anomalies remain an important contributor to adverse pregnancy outcomes in tertiary care settings. Comprehensive antenatal screening, timely referral, adequate periconceptional folic acid supplementation, and inclusion of medically terminated pregnancies in congenital anomaly surveillance programs are essential to improve early diagnosis, counselling, and preventive strategies.

## Introduction

Congenital anomalies are structural or functional abnormalities that occur during intrauterine life and represent a major global public health challenge [1]. They are an important cause of fetal loss, neonatal morbidity, long-term disability, and under-five mortality [2]. According to the World Health Organization, nearly 6% of babies worldwide are born with congenital anomalies, with a disproportionately greater burden in low- and middle-income countries due to limited access to preconception care, periconceptional folic acid supplementation, routine antenatal anomaly screening, prenatal diagnosis, genetic counselling where indicated, and timely referral to specialized fetal and neonatal care services [3].

In India, congenital anomalies contribute substantially to perinatal mortality and childhood disability, although their reported prevalence varies across regions and healthcare settings [4,5]. A national meta-analysis estimated a pooled prevalence of 184.48 per 10,000 births, while another Indian cohort reported congenital anomalies in approximately one out of every 44 births [6]. However, available data remain heterogeneous because many studies are restricted to live births and do not consistently include stillbirths or pregnancies terminated following prenatal diagnosis of fetal anomalies [7]. Tertiary care referral centers, which manage a large number of high-risk pregnancies, provide an opportunity to obtain a more comprehensive estimate of the burden and spectrum of congenital anomalies [8].

Congenital anomalies encompass a broad spectrum of abnormalities involving the central nervous, cardiovascular, gastrointestinal, musculoskeletal, genitourinary, craniofacial, respiratory, and chromosomal systems [9]. Their occurrence and distribution may be influenced by maternal demographic and obstetric factors, including maternal age, parity, antenatal care utilization, gestational age at diagnosis, folic acid supplementation, maternal comorbidities, medication exposure, previous spontaneous abortions, infections during pregnancy, and family history of congenital anomalies [10-14]. Systematic documentation of these characteristics, along with the pattern of congenital anomalies and pregnancy outcomes, is essential for planning preventive strategies, improving prenatal diagnosis, optimizing antenatal counselling, and strengthening perinatal care for affected pregnancies and newborns [15,16]. The present study aimed to determine the prevalence and pattern of congenital anomalies and to describe the maternal demographic and obstetric characteristics, mode of anomaly detection, and pregnancy outcomes among deliveries and medically terminated pregnancies at a tertiary care center.

## Materials and methods

This prospective observational study was conducted in the Department of Obstetrics and Gynaecology, Adichunchanagiri Institute of Medical Sciences (AIMS), Adichunchanagiri University, B.G. Nagara, Karnataka, India, over a period of 12 months from February 2025 to January 2026. The study was undertaken to determine the prevalence and pattern of congenital anomalies among deliveries and medically terminated pregnancies at a tertiary care center. Ethical approval was obtained from the Institutional Ethics Committee of Adichunchanagiri Institute of Medical Sciences, B.G. Nagara, India, before commencement of the study (Approval No. AIMS/IEC/264/2025, dated: 01-01-2025). Written informed consent was obtained from all eligible participants, and confidentiality of patient information was maintained throughout the study. All deliveries occurring during the study period, including live births, stillbirths, and pregnancies undergoing medical termination for documented fetal congenital anomalies, were considered for inclusion in the study. Thus, all eligible cases meeting the selection criteria during the study period were included, and no formal sample size calculation was performed.

All women delivering at the institution during the study period, including live births, stillbirths, and pregnancies undergoing medical termination for documented fetal congenital anomalies, were screened for eligibility. Congenital anomalies were identified primarily through routine antenatal ultrasonography and confirmed by postnatal clinical examination and/or appropriate imaging where required. Prenatal genetic testing was not performed routinely and was undertaken only when clinically indicated and available. Congenital anomalies detected either antenatally or confirmed at the time of delivery were included in the study. Pregnancies with incomplete anomaly-related documentation, women unwilling to participate, and cases in which antenatally suspected anomalies were not confirmed after delivery or postnatal evaluation were excluded.

Information collected included maternal demographic characteristics such as age, parity, residence, antenatal care status, gestational age at diagnosis, and plurality. Obstetric and maternal characteristics, including previous spontaneous abortion, folic acid supplementation, medication exposure, hypertensive disorders of pregnancy, intrauterine infections, polyhydramnios, oligohydramnios, IgA nephropathy, twin pregnancy, previous congenital anomaly, and family history of congenital anomalies, were recorded. Antenatal records and ultrasonography reports were reviewed to determine the mode and timing of anomaly detection. Congenital anomalies were documented and classified according to the organ system involved, and pregnancy outcomes, including medical termination of pregnancy, stillbirth, and live birth, were recorded.

The primary outcome of the study was to determine the prevalence of congenital anomalies among all deliveries and medically terminated pregnancies during the study period. The secondary outcomes included the distribution of congenital anomalies according to the organ system involved, maternal demographic and obstetric characteristics, mode of anomaly detection, and pregnancy outcomes. Data were entered into Microsoft Excel (Microsoft Corporation, Redmond, WA, USA) and analyzed using IBM SPSS Statistics for Windows, Version 26.0 (IBM Corp., Armonk, NY, USA). Categorical variables were summarized as frequencies and percentages and are presented in tables. As the study was descriptive in nature, no inferential analyses or multivariable regression models were performed to evaluate independent associations between maternal risk factors and congenital anomalies.

## Results

Congenital anomalies were identified in 47 (2.5%) of the 1,878 deliveries and medically terminated pregnancies screened during the study period, while the remaining 1,831 (97.5%) pregnancies had no documented congenital anomalies and were not included in further descriptive analyses (Table 1).

**Table 1:** Prevalence of congenital anomalies among the study population (N = 1,878) Data are presented as n (%). Congenital anomalies were identified among the study population of 1,878 deliveries and medically terminated pregnancies. N, total number of pregnancies.

Finding	n (%)
Congenital anomalies detected	47 (2.5%)
No congenital anomalies	1,831 (97.5%)

Among the 47 pregnancies with congenital anomalies, the largest proportion of mothers belonged to the 26-30 years age group (16 (34.0%)), followed by the 21-25 years age group (13 (27.6%)). Multigravida women constituted 28 (59.6%) cases, whereas 19 (40.4%) were primigravida. Congenital anomalies were most frequently diagnosed between 21 and 25 weeks of gestation (28 (59.6%)). Most pregnancies were singleton (46 (97.9%)), had received antenatal care (44 (93.6%)), and were from rural areas (28 (59.6%)). The distribution of maternal age groups among the overall study population was not evaluated because the study was designed to describe pregnancies affected by congenital anomalies (Table 2).

**Table 2:** Maternal characteristics of pregnancies with congenital anomalies (N = 47) Data are presented as n (%). Maternal demographic and obstetric characteristics of pregnancies with congenital anomalies are summarized. N, total number of pregnancies with congenital anomalies.

Variable	Category	n (%)
Age (years)	18-20	3 (6.4%)
	21-25	13 (27.6%)
	26-30	16 (34.0%)
	31-35	11 (23.4%)
	>35	4 (8.5%)
Parity	Primigravida	19 (40.4%)
	Multigravida	28 (59.6%)
Gestational age at diagnosis	<16 weeks	7 (14.9%)
	16-20 weeks	12 (25.5%)
	21-25 weeks	28 (59.6%)
Plurality	Singleton	46 (97.9%)
	Multiple	1 (2.1%)
Antenatal care	Yes	44 (93.6%)
	No	3 (6.4%)
Residence	Urban	19 (40.4%)
	Rural	28 (59.6%)

Prenatal ultrasonography detected congenital anomalies in 37 (78.7%) pregnancies, whereas 10 (21.3%) anomalies were diagnosed only at delivery or during postnatal examination. No congenital anomalies were diagnosed solely by prenatal genetic testing (Table 3).

**Table 3:** Mode of detection of congenital anomalies (N = 47) Data are presented as n (%). The table shows the mode of detection of congenital anomalies among affected pregnancies. N, total number of pregnancies with congenital anomalies.

Mode of detection	n (%)
Detected by prenatal ultrasonography	37 (78.7%)
Detected at delivery/postnatal examination	10 (21.3%)

Among the pregnancies affected by congenital anomalies, 31 (66.0%) underwent medical termination of pregnancy, 10 (21.3%) resulted in stillbirth, and 6 (12.7%) resulted in live birth (Table 4).

**Table 4:** Pregnancy outcomes among pregnancies with congenital anomalies Data are presented as n (%). The table summarizes pregnancy outcomes among pregnancies with congenital anomalies. N, total number of pregnancies with congenital anomalies.

Outcome	n (%)
Medical termination of pregnancy	31 (66.0%)
Stillbirth	10 (21.3%)
Live birth	6 (12.7%)

Congenital anomalies involving the central nervous system were the most common, accounting for 16 (34.0%) cases, followed by circulatory system and gastrointestinal system anomalies, each comprising 10 (21.3%) cases. Musculoskeletal anomalies were observed in 4 (8.5%) cases, while urinary, cleft lip and/or palate, and chromosomal anomalies each accounted for 2 (4.3%) cases. Respiratory system anomalies were identified in 1 (2.1%) case (Table 5).

**Table 5:** Distribution of congenital anomalies according to organ system (N = 47) Data are presented as n (%). The table shows the distribution of congenital anomalies according to the affected organ system. N, total number of pregnancies with congenital anomalies.

Organ system	n (%)
Central nervous system	16 (34.0%)
Circulatory system	10 (21.3%)
Gastrointestinal system	10 (21.3%)
Musculoskeletal system	4 (8.5%)
Urinary system	2 (4.3%)
Cleft lip and/or palate	2 (4.3%)
Chromosomal anomalies	2 (4.3%)
Respiratory system	1 (2.1%)

Among central nervous system anomalies, anencephaly was the most frequently observed anomaly (3 (6.4%)), while spina bifida, hydrocephalus, meningocele, and ventriculomegaly were each reported in 2 (4.3%) cases. Ventricular septal defect was the most common circulatory anomaly (3 (6.4%)), followed by patent ductus arteriosus (2 (4.3%)). Gastrointestinal anomalies were predominantly represented by diaphragmatic hernia, gastroschisis, anorectal atresia, and omphalocele, each occurring in 2 (4.3%) cases. The remaining anomalies across the musculoskeletal, urinary, craniofacial, chromosomal, and respiratory systems were recorded in one or two cases each (Table 6).

**Table 6:** Pattern of congenital anomalies among pregnancies with congenital anomalies (N = 47) Data are presented as n (%). The table presents the pattern of specific congenital anomalies classified according to the affected organ system. N, total number of pregnancies with congenital anomalies; CNS, central nervous system; PDA, patent ductus arteriosus; VSD, ventricular septal defect.

Organ system	Congenital anomaly	n (%)
Central nervous system	Anencephaly	3 (6.4%)
	Spina bifida	2 (4.3%)
	Hydrocephalus	2 (4.3%)
	Meningocele	2 (4.3%)
	Arnold-Chiari malformation type II	1 (2.1%)
	Dandy-Walker malformation	1 (2.1%)
	Ventriculomegaly	2 (4.3%)
	Aqueductal stenosis	1 (2.1%)
	Choroid plexus cyst	1 (2.1%)
	Meningomyelocele	1 (2.1%)
Circulatory system	Ventricular septal defect	3 (6.4%)
	Patent ductus arteriosus	2 (4.3%)
	Tetralogy of Fallot	1 (2.1%)
	Coarctation of aorta	1 (2.1%)
	Hypoplastic left heart syndrome	1 (2.1%)
	Complete heart block	1 (2.1%)
	Supraventricular tachycardia	1 (2.1%)
Gastrointestinal system	Diaphragmatic hernia	2 (4.3%)
	Gastroschisis	2 (4.3%)
	Anorectal atresia	2 (4.3%)
	Omphalocele	2 (4.3%)
	Tracheoesophageal fistula	1 (2.1%)
	Vitellointestinal duct anomaly	1 (2.1%)
Musculoskeletal system	Bilateral congenital talipes equinovarus	2 (4.3%)
	Duchenne muscular dystrophy	1 (2.1%)
	Skeletal dysplasia	1 (2.1%)
Urinary system	Dilated pelvicalyceal system	1 (2.1%)
	Ectopic kidney	1 (2.1%)
Cleft lip and/or palate	Cleft lip with cleft palate	1 (2.1%)
	Cleft lip	1 (2.1%)
Chromosomal anomalies	Down syndrome	1 (2.1%)
	Edwards syndrome	1 (2.1%)
Respiratory system	Congenital pulmonary airway malformation	1 (2.1%)

Previous spontaneous abortion was the most frequently reported maternal or obstetric characteristic, occurring in 26 (55.3%) pregnancies with congenital anomalies. Lack of folic acid intake was documented in 4 (8.5%) pregnancies, while a history of drug intake was reported in 3 (6.4%). Family history of congenital anomalies, oligohydramnios, hypertensive disorders of pregnancy, intrauterine infections, and polyhydramnios were each recorded in 2 (4.3%) pregnancies. IgA nephropathy, previous congenital anomaly, and twin pregnancy were each recorded in 1 (2.1%) pregnancy (Table 7).

**Table 7:** Maternal and obstetric characteristics among pregnancies with congenital anomalies (N = 47) Data are presented as n (%). The table summarizes maternal and obstetric characteristics observed among pregnancies with congenital anomalies. N, total number of pregnancies with congenital anomalies; IgA, immunoglobulin A.

Characteristic	n (%)
Previous spontaneous abortion	26 (55.3%)
Lack of folic acid intake	4 (8.5%)
History of drug intake	3 (6.4%)
Family history of congenital anomalies	3 (6.4%)
Oligohydramnios	2 (4.3%)
Hypertensive disorders of pregnancy	2 (4.3%)
Intrauterine infections	2 (4.3%)
Polyhydramnios	2 (4.3%)
IgA nephropathy	1 (2.1%)
Previous congenital anomaly	1 (2.1%)
Twin pregnancy	1 (2.1%)

## Discussion

The present study reported a prevalence of congenital anomalies of 47 (2.5%) among 1,878 deliveries and medically terminated pregnancies at a tertiary care referral center. This estimate is comparable to that reported by Rathod and Samal, who observed 140 (2.28%) congenital anomalies among 6,134 deliveries, suggesting a similar burden in hospital-based Indian populations [12]. A higher prevalence of 86 (5.49%) anomalies among 1,565 pregnancies was reported by Timilsina and Gupta, which may be attributable to differences in referral patterns and a greater proportion of pregnancies terminated following prenatal diagnosis of fetal anomalies [10]. In contrast, the substantially lower prevalence reported by Kale et al. reflects population-based surveillance that primarily included live births and is therefore likely to underestimate the true burden [17]. Likewise, Kumar et al. reported 1,578 (1.82%) congenital anomalies over two decades of institutional surveillance [8]. Collectively, these findings indicate that hospital-based studies incorporating antenatally diagnosed and medically terminated pregnancies provide a more comprehensive estimate of the prevalence of congenital anomalies.

The maternal profile observed in the present study was largely comparable to previous reports. Most anomaly-affected pregnancies occurred among women aged 26-30 years (16 (34.0%)), multigravida mothers (28 (59.6%)), and those residing in rural areas (28 (59.6%)), while most anomalies were diagnosed between 21 and 25 weeks of gestation (28 (59.6%)) despite high antenatal care utilization (44 (93.6%)). Similar observations were reported by Timilsina and Gupta, who documented predominance among mothers aged 26-30 years (30 (34.88%)), multiparous women (52 (60.47%)), and rural residents (51 (59.30%)) [10]. Rathod and Samal also observed a greater proportion of anomalies among younger mothers, particularly those aged 21-25 years (59 (42.1%)), and multigravida women (85 (60.71%)), whereas Kale et al. demonstrated a higher prevalence at the extremes of maternal age in population-based surveillance [12,17]. Nearly four-fifths of congenital anomalies in the present study were detected by prenatal ultrasonography (37 (78.7%)), closely corresponding to the antenatal detection rate reported by Timilsina and Gupta (68 (79.0%)), highlighting the important role of routine anomaly scans in facilitating prenatal diagnosis [10]. However, postnatal detection in (10 (21.3%)) cases indicates that certain congenital defects remain difficult to identify during antenatal screening.

The distribution of congenital anomalies in the present study demonstrated that central nervous system anomalies were the most frequent (16 (34.0%)), followed by circulatory (10 (21.3%)) and gastrointestinal anomalies (10 (21.3%)). A similar system-wise distribution has been consistently reported in tertiary care studies by Timilsina and Gupta, in which central nervous system anomalies accounted for (29 (33.72%)), followed by circulatory (19 (22.10%)) and gastrointestinal anomalies (18 (20.94%)) [10]. Rathod and Samal also identified central nervous system anomalies as the predominant category (40 (28.5%)) [12]. At the individual anomaly level, anencephaly (3 (6.4%)) was the most common neural tube defect, while ventricular septal defect (3 (6.4%)) was the most frequent cardiac anomaly. These observations are consistent with those reported by Timilsina and Gupta and Rathod and Samal, whereas Kumar et al. reported a relatively greater contribution of cardiac anomalies, possibly reflecting differences in postnatal diagnostic facilities and case ascertainment [8,10,12]. The similarity of findings across tertiary referral centers suggests that neural tube defects continue to constitute a major proportion of congenital anomalies despite advances in antenatal screening and preventive strategies.

Pregnancy outcomes in the present study showed that 31 (66.0%) pregnancies underwent medical termination, 10 (21.3%) resulted in stillbirth, and 6 (12.8%) resulted in live birth. This pattern is comparable to that reported by Timilsina and Gupta, in which 56 (65.0%) pregnancies underwent termination, 28 (32.6%) resulted in live births, and 2 (2.4%) resulted in stillbirth [10]. Hospital-based studies that include medically terminated pregnancies therefore provide a more complete representation of the burden of congenital anomalies than studies restricted to live births alone. Previous spontaneous abortion was the most frequently documented maternal characteristic (26 (55.3%)), whereas lack of folic acid supplementation (4 (8.5%)), medication exposure (3 (6.4%)), hypertensive disorders of pregnancy (2 (4.3%)), oligohydramnios (2 (4.3%)), polyhydramnios (2 (4.3%)), intrauterine infections (2 (4.3%)), and family history of congenital anomalies (2 (4.3%)) were observed less frequently. Similar observations regarding previous pregnancy loss and folic acid supplementation have been reported by Timilsina and Gupta, while Rathod and Samal additionally emphasized the contribution of consanguinity and gestational diabetes mellitus, reflecting regional differences in maternal risk factors [10,12]. Because the present study was descriptive in nature, no multivariable analyses were performed to evaluate independent associations between these maternal characteristics and congenital anomalies. Furthermore, information on gestational diabetes mellitus and certain maternal infections was not systematically available for all participants and therefore could not be analyzed separately.

Strengths and limitations

The findings of the present study emphasize the importance of strengthening preconception care, ensuring adequate periconceptional folic acid supplementation, promoting timely antenatal anomaly screening, and improving referral pathways for pregnancies complicated by congenital anomalies. Inclusion of medically terminated pregnancies in congenital anomaly surveillance programs may provide a more accurate estimate of disease burden and facilitate planning of preventive and prenatal diagnostic services. This study has certain limitations. Being a single-center tertiary care study, the findings may not be generalizable to the wider community, and referral bias may have increased the proportion of severe congenital anomalies. In addition, incomplete clinical documentation in some cases and the inability to assess long-term neonatal outcomes may have influenced the overall findings. Moreover, the descriptive study design did not permit assessment of independent maternal risk factors, and information regarding gestational diabetes mellitus and selected maternal infections was not consistently available for analysis.

## Conclusions

Congenital anomalies represent an important cause of adverse fetal and neonatal outcomes in tertiary care settings. Central nervous system anomalies were the most frequently encountered, followed by circulatory and gastrointestinal anomalies. Most anomalies were detected during antenatal ultrasonography, facilitating timely diagnosis and appropriate pregnancy management. Previous spontaneous abortion was the most commonly observed maternal characteristic among affected pregnancies. These findings emphasize the importance of strengthening preconception care, ensuring adequate periconceptional folic acid supplementation, promoting routine antenatal anomaly screening, and maintaining comprehensive congenital anomaly surveillance, including medically terminated pregnancies, to support early diagnosis, appropriate counselling, timely referral for multidisciplinary fetal and neonatal care, and optimized management of affected pregnancies and newborns.
